# High Accommodative Convergence/Accommodation Ratio Consecutive Esotropia Following Surgery for Intermittent Exotropia: Clinical Feature, Diagnosis, and Treatment

**DOI:** 10.3390/jcm10102135

**Published:** 2021-05-15

**Authors:** Byung Joo Lee, Hyun Taek Lim

**Affiliations:** Asan Medical Center, Department of Ophthalmology, University of Ulsan College of Medicine, Seoul 05505, Korea; ozma805@gmail.com

**Keywords:** consecutive esotropia, high accommodative convergence/accommodation ratio, intermittent exotropia

## Abstract

Consecutive esotropia is a common and stereopsis-threatening consequence of surgery for intermittent exotropia. However, too little attention has been paid to the accommodative convergence per accommodation (AC/A) ratio in this condition. We aimed to describe the clinical features of patients who developed consecutive esotropia with a high AC/A following surgery for intermittent exotropia, compared to those with normal AC/A. In this retrospective cohort study, we identified 54 patients with consecutive esotropia who remained esotropic at one month after surgery. Patients were divided into two groups: normal AC/A and high AC/A groups. Clinical features and outcomes were compared between the two groups. Fourteen (25.9%) of the 54 were classified as high AC/A consecutive esotropia. Good preoperative control at near was the only significant preoperative factor associated with the development of high AC/A consecutive esotropia. Bifocal glasses were prescribed for all patients with high AC/A consecutive esotropia, and 11 (78.6%) of them achieved satisfactory alignment at distance and near fixations using bifocals. Patients with high AC/A consecutive esotropia had a significantly more successful alignment (0.9 vs. 13.0 prism diopters, *p* < 0.001) and better stereopsis (67.9 vs. 670.0 arc seconds, *p* = 0.04) than the normal AC/A counterparts at the final follow-up. We suggest that high AC/A consecutive esotropia could be successfully managed by wearing bifocals in most cases. A high AC/A ratio in patients with consecutive esotropia may be considered as a clinical marker heralding a better prognosis.

## 1. Introduction

The difference in distance and near deviation has long been of interest in characterizing the phenotypic spectrum of intermittent exotropia [1,2]. A group of patients with intermittent exotropia may have smaller exodeviation at near than at distance by 10 prism diopters (PD) or more, even after performing the prolonged monocular occlusion test; this type of exotropia is called true divergence-excess exotropia. It is well accepted that a significant proportion of patients with true divergence-excess exotropia may have a high accommodative convergence/accommodation (AC/A) ratio [1,3]. Brodsky and Fray have reported that the high AC/A ratio could persist even after surgical correction of the divergence-excess intermittent exotropia [4]. In addition, Kushner has found that nearly all patients with intermittent exotropia with a high AC/A ratio develop consecutive esotropia after exotropia surgery if the surgery is performed based on distance deviation [5].

Consecutive esotropia occurs in up to 20% of patients following surgery for IXT. A transient postoperative esotropia is desirable because it helps to maintain long-term orthophoria with good stereopsis. However, if the postoperative esodeviation persists for more than 1 month, it is likely to result in consecutive esotropia [6]. High AC/A ratio, lateral incomitancy, immediate postoperative overcorrection exceeding 17 PD, and younger age at surgery are risk factors predisposing to consecutive esotropia [7,8]. Although consecutive esotropia is a common clinical consequence in strabismus practice, the AC/A ratio in consecutive esotropia has not received the proper attention it deserves in the literature. Over the past few years, we have encountered pediatric patients with high AC/A ratio consecutive esotropia in our clinical practice. The primary purpose of this study is to describe the clinical spectrum and features of high AC/A consecutive esotropia following surgery for IXT. The secondary aim is to compare preoperative and postoperative clinical characteristics between high AC/A and normal AC/A consecutive esotropia patients. 

## 2. Methods

### 2.1. Subjects

We retrospectively reviewed the medical records of all pediatric patients aged under 18 who underwent surgery for intermittent exotropia between January 2013 and December 2019 at the Asan Medical Center, University of Ulsan College of Medicine in Seoul, Korea. For this study, we included only children who underwent bilateral lateral rectus (BLR) recession by a single surgeon (H.T.L.). Patients were excluded if they had A or V pattern strabismus, lateral incomitancy, associated hypertropia ≥ 6 PD, oblique dysfunction of +2 or more, any degree of dissociated vertical deviation, amblyopia (≥2 lines interocular difference or best-corrected visual acuity worse than 0.2 logMAR), significant ocular or neurologic disorders other than strabismus, and history of previous strabismus surgery. Patients with a shorter than 12 months of postoperative follow-up were also excluded. Among these patients included in this study, we further identified a group of patients who developed consecutive esotropia following BLR recession. Consecutive esotropia was defined as residual manifest esodeviation of ≥10 prism diopters (PD) at 1 month after surgery. This study was approved by the Institutional Review Board of the Asan Medical Center (IRB No. 2020-0327) and was conducted in accordance with the Declaration of Helsinki. 

### 2.2. Preoperative-Assessment Data

The following preoperative clinical data were retrieved from medical records: age at presentation, age at surgery, gender, angle of deviation at distance and near fixation, stereoacuity, and degree of control. Stereoacuity was measured by the Titmus test in all children aged 4 years or older. The degree of control was assessed by the LACTOSE scoring system at near and distance fixation [9]. The exodeviation angle was measured using the prism and alternate cover test (PACT) with accommodative targets at 1/3 and 6 m. A full spectrum of refractive error corrections was recommended if the patient had hyperopia ≥ +2.00 D, myopia ≤ −1.0 D, or astigmatism ≥ 1.50 D.

### 2.3. Surgical Procedures

All surgical procedures conducted in this study were symmetric BLR recession. The surgical amount was determined based on maximal deviation and degree of control. With that amount of surgery, we aimed to achieve an ideal immediate postoperative target angle. Our ideal postoperative target range was 4 to 10 PD of esotropia 1 week after surgery at the first postoperative visit.

### 2.4. Postoperative-Assessment Data

Patients were invariably examined at postoperative 1 week and were followed up at 1, 6, 12, and 24 months later. The angle of near and distance deviation was measured by PACT at each postoperative visit. If the diagnosis of consecutive esotropia was made at 1 month after BLR recession, the patients underwent cycloplegic refraction, and full spectacle corrections were prescribed with 2 h of daily patching therapy. In case the esotropia persisted at the 6-month postoperative visit, prism glasses were prescribed. Whenever the patients with consecutive esotropia showed near deviation greater than distance deviation by 10 PD or more, we routinely performed a +3.00 diopter (D) lens test at near fixation [8]. If the near esodeviation decreased more than 10 PD by placing +3.00 diopter lenses in front of the eyes, then the diagnosis of high AC/A consecutive esotropia was made for those patients. AC/A ratio was measured with the lens gradient method at near (33 cm) using +3.0 D lenses after a 1-h monocular occlusion. In all patients diagnosed with high AC/A consecutive esotropia, we prescribed pupil-bisecting bifocal glasses with +3.0 D near add. In high AC/A ratio consecutive esotropia patients who are wearing bifocals, ocular patching (2 h per day) was recommended if they had distance esotropia greater than 10 prism diopters even after spectacle correction.

### 2.5. Statistical Analysis

All statistical analyses were performed using PASW statistical software (Ver. 21 for Windows, SPSS, Inc., Chicago, IL, USA). Fisher’s exact tests were used to compare clinical characteristics between study groups. Mann–Whitney U tests were applied to compare the mean values of clinical variables. Statistical significance was defined as *p* < 0.05.

## 3. Results

**Figure 1:**
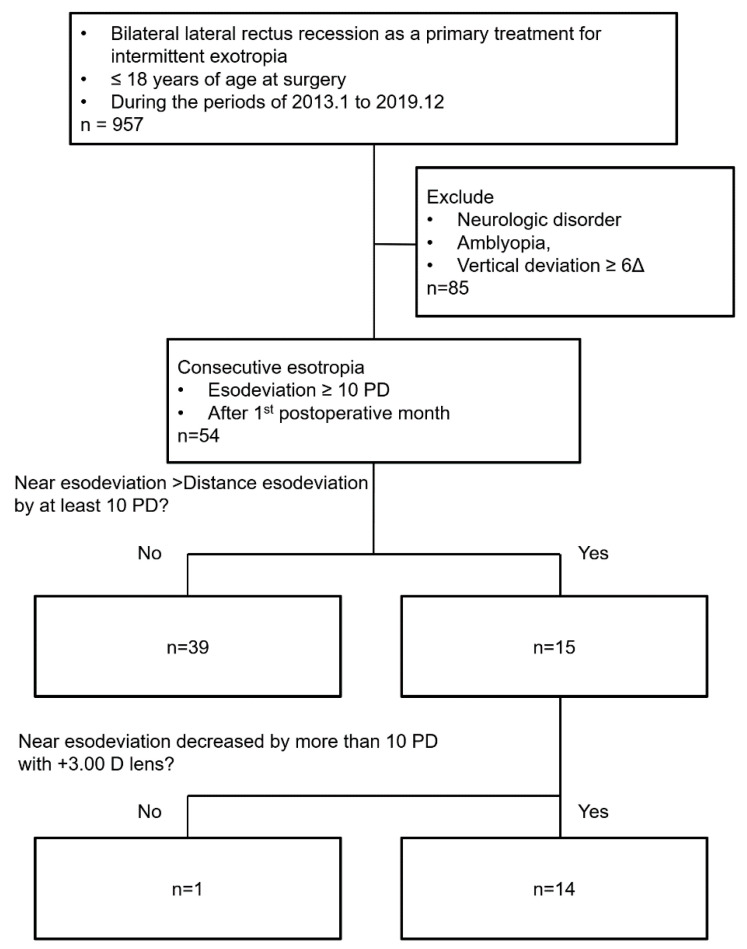
A flow chart for identifying patients with high accommodative convergence/accommodation (AC/A) ratio consecutive esotropia.

We reviewed the medical records of 957 pediatric patients (<18 years of age) with intermittent exotropia who underwent BLR recession for surgical treatment. Among those, 85 were noted to have neurological disorders, amblyopia, and associated vertical deviation ≥ 6 PD, and were excluded from the study. Out of the remaining 872 patients, 805 (92.3%) developed esodeviation at either distance or near 1 week after surgery. With time, 1 month after surgery, only 54 (6.2%) remained esotropic and were identified as consecutive esotropia following surgery according to our aforementioned diagnostic criteria. Of those, 15 (27.8%) had an esodeviation greater at near than distance by ≥10 PD. Using +3.00 D near add, the angle of esodeviation decreased by more than 10 PD close to the distance deviation in all but one patient (Figure 1). Hence, among the 54 patients diagnosed with consecutive esotropia, 14 were classified as high AC/A ratio consecutive esotropia, and the remaining 40 were classified as normal AC/A consecutive esotropia. The normal AC/A consecutive esotropia group included 1 having non-accommodative convergence excess consecutive esotropia and the other 39 patients having basic type consecutive esotropia. 

Out of the 54 patients with consecutive esotropia, 36 (66.7%) were female. Mean ages at presentation and at the time of surgery for intermittent exotropia were 4.1 ± 2.8 and 5.4 ± 2.5 years, respectively. All 54 patients were determined preoperatively as having basic type exotropia. We compared preoperative clinical characteristics between patients with high AC/A consecutive esotropia and normal AC/A consecutive esotropia (Table 1). None had low AC/A consecutive esotropia. There were no significant differences in the age at presentation, age at surgery, gender, type of exotropia, amount of exotropia, refractive errors, and stereoacuity between the two groups. However, with regard to the degree of control, a relatively good preoperative control (LACTOSE score 0 to 2) at near was significantly associated with the development of postoperative high AC/A consecutive esotropia (*p* = 0.03; Fisher’s exact test), whereas the good preoperative control at distance (LACTOSE score 0 to 2) was not the case (*p* = 0.50; Fisher’s exact test).

**Figure 2:**
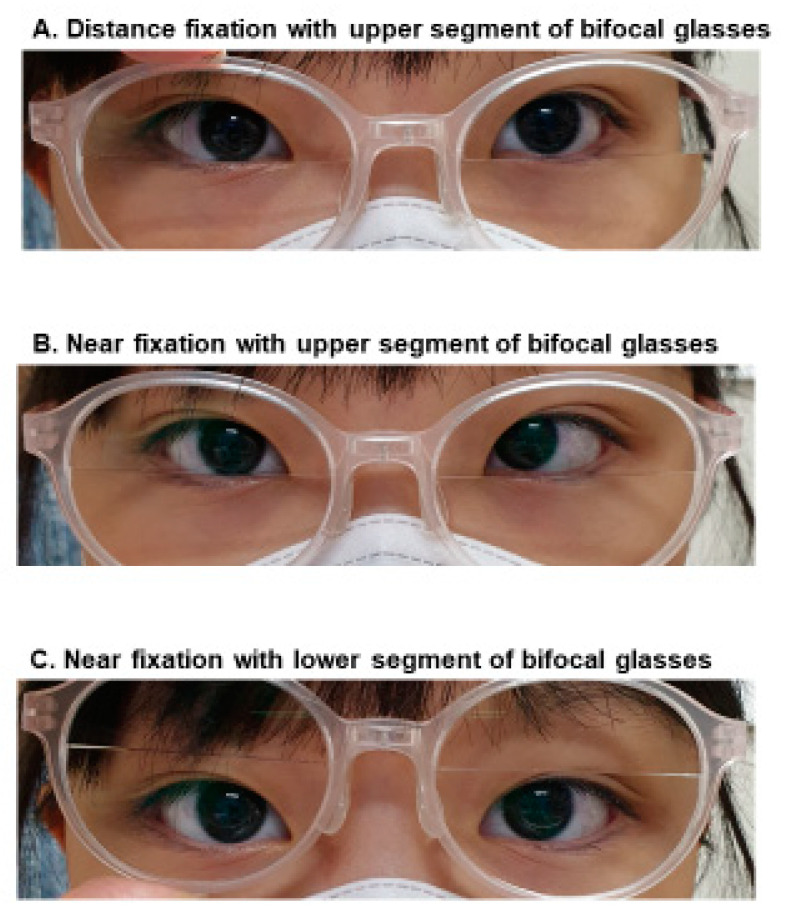
Representative photographs of a high accommodative convergence/accommodation (AC/A) ratio consecutive esotropia patient. Ocular alignment at distance fixation using the upper segment of bifocal glasses (upper), at near fixation using the upper segment of bifocal glasses (middle), and at near fixation using the lower segment of bifocal glasses (lower).

The clinical features of patients with high AC/A consecutive esotropia are summarized in Table 2. None of the patients preoperatively showed a significant hypermetropia (>+2.00 D). The mean value of the AC/A ratio measured postoperatively by the gradient method at near fixation was 6.5 ± 2.4 (ranged from 4.3 to 13.3). All patients were followed for at least 12 months postoperatively, and the mean postoperative follow-up duration was 37.4 ± 16.4 months. Bifocal glasses were prescribed for all 14 patients with high AC/A consecutive esotropia for a mean duration of 18.6 months (median 16.1 months). Eleven of the 14 patients achieved orthophoric alignment at near fixation with bifocal glasses. Figure 2 shows a representative case in which near esodeviation was fully corrected with bifocal glasses. Five of the 11 patients could be successfully weaned from the bifocals after a 25-month median duration of wearing bifocals. Another 5 of the 11 patients needed to continue using bifocal glasses to control the near esodeviation. The mean duration of wearing bifocals in patients who could not wean off bifocals (median 7.5 months and mean 11 months) was significantly shorter than that of the successfully weaned patients (median 25.0 months and mean 31.6 months) with statistical significance (*p* = 0.03). The remaining 1 of the 11 patients showed a satisfactory alignment for the first 12 months of wearing bifocals, but later with time, the esotropia has worsened to decompensated esotropia, which required surgical treatment. In 3 of the 14 patients, bifocal glasses could provide only a partial correction of esotropia. Two of them underwent surgery (bilateral medial rectus recession) for the correction of residual esotropia, and one of them wanted to continue wearing bifocal glasses. In this series, 4 out of 5 patients successfully weaned bifocals after 8 years of age (range from 8 to 11 years). Patients who could not wean the bifocals were 6 to 8 years old at last follow-up.

Overall, 3 (21.4%) patients underwent surgical treatment for the residual esotropia in the high AC/A group, while 11 (27.5%) patients did so in the normal AC/A group. When comparing postoperative characteristics between high AC/A and normal AC/A consecutive esotropia groups, there were no significant differences in the amount of esodeviation at the first postoperative month and reoperation rate. However, in the aspects of the final motor and sensory outcome, the high AC/A consecutive esotropia group showed significantly better outcomes than the normal AC/A consecutive esotropia group. The high AC/A esotropia patients could achieve more successful alignment and better stereopsis at the final follow-up assessment (Table 1).

## 4. Discussion

In this study, we have demonstrated that among the patients with consecutive esotropia following bilateral rectus muscle recession for intermittent exotropia, some patients show near-distance disparity esotropia, most of which are associated with a high AC/A ratio. According to our results, approximately one-fourth of consecutive esotropia patients (14 out of 54) demonstrated a high AC/A feature. Although the definition of consecutive esotropia varies between studies, the reported incidence of consecutive esotropia following exotropia surgery ranges from 4.94% to 20% [10,11,12,13]. In our series, the incidence of consecutive esotropia is comparable with previous reports.

High AC/A ratio is a well-documented clinical feature associated with a larger near deviation in patients with non-refractive accommodative esotropia. However, it has become apparent that this high AC/A ratio can also be found in some patients with intermittent exotropia [4,5,14]. Previously reported high AC/A ratio incidence among patients with divergence-excess exotropia ranged from 5% to 21% [1,14,15]. Earlier studies investigating the effect of conventional strabismus surgery on the AC/A ratio have shown conflicting results. Lucas et al. have suggested that strabismus surgery involving the medial rectus could decrease the AC/A ratio in esotropia and exotropia patients [16]. In contrast, several studies on the high AC/A ratio associated with intermittent exotropia showed that the AC/A ratio does not change even after surgery for exotropia [4,17]. Kushner also has reported that patients with intermittent exotropia having high AC/A invariably shows postoperative esotropia with persistent high AC/A [14]. 

Despite the common occurrence of consecutive esotropia, very few studies have paid attention to the role of the AC/A ratio in the development of consecutive esotropia after surgery for intermittent exotropia. Furthermore, to the best of our knowledge, no studies have been conducted to entirely focus on the AC/A ratio in patients with consecutive esotropia. The present study described 14 children who developed high AC/A consecutive esotropia after intermittent exotropia surgery. All of the patients showed a favorable response to bifocal glasses for at least the first few months, mostly for a longer time. In most cases, the esodeviation at near was remarkably decreased to less than 5 PD by wearing bifocals. We think it is worthwhile to note that children with high AC/A consecutive esotropia finally achieved better alignment and stereopsis compared with normal AC/A counterparts. It may be inferred from this finding that a high AC/A ratio can be considered a feature associated with a better prognosis in patients with consecutive esotropia.

Given its common association with a feature of distance-near disparity, it is of considerable interest that the high AC/A ratio can be discovered postoperatively in patients with basic type intermittent exotropia without apparent distance-near disparity. Unfortunately, we did not routinely measure the AC/A ratio for the patients with basic type intermittent exotropia. All 14 patients who developed high AC/A consecutive esotropia in our study had distance deviations equal to or even smaller than near deviations preoperatively. Under these circumstances, we did not expect the patients to carry a high AC/A ratio; thus, the AC/A ratio was not measured preoperatively for these patients. It is uncertain whether our patients with high AC/A consecutive esotropia had a high AC/A or normal AC/A ratio preoperatively. However, from our results, it would be reasonable to assume that even patients with basic intermittent exotropia may have a high AC/A ratio when measured preoperatively. If it is the case, it seems highly likely that the high AC/A ratio would persist even after surgery and thereby underlie the development of an esodeviation greater at near than distance, as shown in our patients. Given that a good control (LACTOSE score 0 to 2) at near was significantly linked to the development of high AC/A consecutive esotropia, patients with intermittent exotropia having good near control may have a higher probability of being high AC/A ratio. The authors postulate that bilateral lateral rectus recession in basic type intermittent exotropia patients with good motor control at near might be related to the risk of postoperative overcorrection especially at near fixation. Future investigation should be ensued to delineate optimal surgical options in these patients.

The difference between distance and near deviation became smaller with time, falling to less than 5 PD, as the esodeviation diminished by treatments in the present study. This finding is in good agreement with previous studies. Lucas et al. showed that the AC/A ratio was significantly reduced after surgery for both esotropic and exotropic patients. Kushner has reported a long-term, time-dependent normalization of the AC/A ratio in patients who developed consecutive esotropia following surgery for high AC/A ratio divergence-excess intermittent exotropia [14]. Besides, Parks found that abnormal AC/A ratio in exotropia patients could be improved even without treatments [18]. AC/A ratio can be assumed to undergo normalization with ages.

Part-time patching and prism glasses might be the two most common non-surgical management options for consecutive esotropia in current practice. We showed that bifocals could be a possible treatment option if the consecutive esotropia is related to a high AC/A ratio. Bifocal prescriptions were beneficial in that most patients treated with bifocal glasses could achieve orthophoric alignment at both distance and near on final follow-up assessments. Moreover, patients with high AC/A ratio became less dependent upon bifocal glasses with age. However, the response to a variety of non-surgical treatments was not always satisfactory, especially in patients with significant residual distance deviation even after wearing bifocals; they required surgery to correct the residual esodeviation. This poor response shown in some patients with high AC/A consecutive esotropia was assumed to be related to the non-accommodative component (esodeviation that is not relaxed by glasses) of their esotropia. Considering the ongoing debates on the influence of bifocal glasses in esotropia patients [19,20], we recommend that further studies should be undertaken to determine the efficacy of wearing bifocals in a larger number of patients with consecutive esotropia.

Our study also had some limitations that need to be pointed out. The major limitations of this study included the retrospective design and the small study population. Due to this study’s retrospective nature, we should say that we could not fully standardize the treatment protocol for consecutive esotropia across the study patients. Nonetheless, since only a single strabismus surgeon managed and followed all included patients according to his best judgment throughout the whole period of study, the influence of this study’s non-standardization issue does not seem to be significant. The current study was also unable to analyze the preoperative AC/A ratio in patients who developed postoperative AC/A ratio. Given the finding of the AC/A ratio being measured to be high postoperatively, it may be assumed that the AC/A ratio should be high preoperatively as well. However, it was an unexpected and noteworthy finding that even patients with basic intermittent exotropia could develop a high AC/A ratio consecutive esotropia. From this finding, we suggest extra caution should be exercised to avoid too much overcorrection when surgery is planned for intermittent exotropia patients with good control at near.

In conclusion, approximately a fourth of the patients who developed consecutive esotropia following BLR recession for intermittent exotropia were found to have a high AC/A ratio. High AC/A consecutive esotropia can develop after surgery in patients having basic type intermittent exotropia without distance-near disparity preoperatively. Most of these patients could be successfully managed by wearing bifocal glasses, although surgical treatment was required in a small proportion of patients remaining with a residual significant distance esodeviation. The children with basic type intermittent exotropia and their parents should be informed preoperatively about the possibility of developing high AC/A ratio consecutive esotropia after surgery for exotropia. High AC/A ratio in patients with consecutive esotropia could be considered as a postoperative feature heralding a better prognosis compared with a normal AC/A ratio.

## Figures and Tables

**Table 1:** Comparison of the pre- and postoperative characteristics between high AC/A and normal AC/A consecutive ET patients.

	High AC/A Ratio Group (*n* = 14)	Normal AC/A Ratio Group (*n* = 40)	*p* Value
Mean age at presentation (years)	3.9 ± 2.1	4.1 ± 3.0	0.89 ^†^
Mean age at surgery (years)	4.9 ± 1.9	5.6 ± 2.7	0.42 ^†^
Postoperative F/U duration (months)	37.4 ± 16.4	28.8 ± 16.6	0.30 ^†^
Onset of consecutive ET after surgery (months)	4.1 ± 5.0	5.0 ± 8.1	0.86 ^†^
Male gender	6 (42.9%)	12 (30%)	0.29 ^‡^
Type of exotropia			
Basic type	14	40	0.71 ^‡^
Divergence excess type	0	0	
Convergence insufficiency type	0	0	
Preoperative control score (LACTOSE) at distance			
0–2	1 (8.3%)	1 (3.2%)	0.50 ^‡^
3–4	11 (91.6%)	30 (96.8%)	
NA	2	10	
Preoperative control score (LACTOSE) at near			
0–2	10 (83.3%)	14 (46.7%)	0.03 ^‡^
3–4	2 (16.6%)	8 (53.4%)	
NA	2	10	
Preoperative SE, OD (D)	−0.6 ± 2.1	−0.4 ± 1.4	0.96 ^†^
Preoperative SE, OS (D)	−0.5 ± 1.8	−0.3 ± 1.4	0.99 ^†^
Preoperative stereoacuity (seconds of arc)			
≤100	8	21	0.36 ^‡^
>100	4	6	
NA	2	13	
Mean	111.7	101.9	0.46
Preoperative angle of exodeviation			
Distance (PD)	27.3 ± 7.5	28.3 ± 6.5	0.34 ^†^
Near (PD)	29.4 ± 7.0	29.7 ± 6.4	0.50 ^†^
Absolute value of distance-near angle (PD)	2.1 ± 2.3	2.8 ± 3.9	0.82 ^†^
Postoperative angle of esodeviation (1st postoperative month)			
Distance (PD)	−7.4 ± 8.1	−9.8 ± 6.5	0.13 ^†^
Near (PD)	−7.4 ± 8.1	−9.6 ± 6.6	0.18 ^†^
Surgical correction for consecutive ET			
Yes	3 (21.4%)	11 (27.5%)	0.45 ^‡^
No	11 (78.6%)	29 (72.5%)	
Final angle of deviation			
Distance (PD)	−0.9 ± 2.4	−13.0 ± 5.0	<0.001 ^†^
Near (PD)	−0.4 ± 1.6	−12.4 ± 5.7	<0.001 ^†^
Final stereoacuity (seconds of arc)			
≤100	13 (92.9%)	17 (46.0%)	0.11 ^‡^
>100	1 (7.1%)	33 (54.0%)	
Mean	67.9	670.0	0.04 ^†^

D = Diopters; DVD = dissociated vertical deviation; ET = esotropia; F/U = follow-up; LACTOSE = Look And Cover, then Ten seconds of Observation Scale for Exotropia; NA = not available; PD = Prism diopters; SE = spherical equivalent; †, Mann–Whitney U test; ‡, Fisher’s exact test. The angle of deviation was represented as positive numbers in exodeviation and negative numbers in esodeviation.

**Table 2:** Preoperative clinical features and final outcomes of patients with high AC/A ratio consecutive esotropia.

Patient No	Sex	Age at Surgery for XT (year)	Preop Angle of XT (Δ)	Preop SE (D)	Preop SA (SecArc)	Surgery	Onset of Consecutive ET (mo)	Max. Angle of Consecutive ET (Δ)	AC/A Ratio	Time from Surgery to Start of Bifocals (mo)	Duration of Wearing Bifocals (mo)	After Wearing Bifocal Glasses	Response to Bifocals	Clinical Course	Postoperative FU Period (mo)	Final Alignment	Final SA (SecArc)
1	F	4	Dcc 25 Ncc 30	R + 1.50 L + 2.00	140	BLRc 6.50	2	Dcc ortho Ncc 30	8.3	4	51	Dcc ortho Ncc 30 Ncc+3 ortho	Good response to bifocals	Successfully weaned from bifocals	74	Dcc ortho Ncc ortho	100
2	F	2	Dcc 50 Ncc 50	R − 0.50 L − 0.50	NA	BLRc 9.00	6	Dcc 5 Ncc 20	6.7	19	10	Dcc ortho Ncc 16 Ncc+3 ortho	Good response to bifocals	Successfully weaned from bifocals	50	Dcc ortho Ncc 5 ET	80
3	M	3	Dcc 30 Ncc 30	R + 1.00 L + 1.00	NA	BLRc 7.25	1	Dcc 20 Ncc 30	4.7	5	54	Dcc ortho Ncc 10 Ncc+3 ortho	Good response to bifocals	Successfully weaned from bifocals	59	Dcc ortho Ncc ortho	80
4	M	8	Dcc 20 Ncc 23	R − 2.25 L − 2.25	40	BLRc 6.50	2	Dcc 10 Ncc 25	6.7	4	25	Dcc ortho Ncc 10 Ncc+3 ortho	Good response to bifocals	Successfully weaned from bifocals	29	Dcc ortho Ncc ortho	40
5	M	7	Dcc 20 Ncc 25	R + 0.50 L + 0.50	80	BLRc 6.25	1	Dcc 10 Ncc 25	5.0	2	18	Dcc ortho Ncc 14 ET Ncc+3 ortho	Good response to bifocals	Successfully weaned from bifocals	32	Dcc ortho Ncc ortho	80
6	F	4	Dcc 30 Ncc 35	R + 0.50 L + 0.50	40	BLRc 7.50	4	Dcc 10 Ncc 30	5.0	5	25	Dcc ortho Ncc 20 ET Ncc+3 ortho	Good response to bifocals	Need to continue wearing bifocals	30	Dcc ortho Ncc 20 ET Ncc+3 ortho	80
7	F	4	Dcc 25 Ncc 30	R + 2.00 L + 2.00	60	BLRc 7.25	1	Dcc 10 Ncc 25	6.7	12	8	Dcc 10 Ncc 25 Ncc+3 ortho	Good response to bifocals	Need to continue wearing bifocals	20	Dcc 5 ET Ncc 20 ET Ncc+3 ortho	40
8	F	6	Dcc 25 Ncc 25	R 0.00 L + 1.00	60	BLRc 7.00	6	Dcc 6 Ncc 30	8.0	9	6	Dcc ortho Ncc 20 ET Ncc+3 ortho	Good response to bifocals	Need to continue wearing bifocals	21	Dcc ortho Ncc 16 ET Ncc+3 ortho	40
9	M	5	Dcc 25 Ncc 25	R − 0.75 L − 0.75	100	BLRc 6.75	1	Dcc 5 Ncc 20	5.0	5	7	Dcc 2 ET Ncc 20 ET Ncc+3 ortho	Good response to bifocals	Need to continue wearing bifocals	12	Dcc ortho Ncc 14 ET Ncc+3 ortho	60
10	F	5	Dcc 25 Ncc 30	R + 1.50 L + 1.75	40	BLRc 7.25	1	Dcc 12 ET Ncc 30 ET	6.0	19	16	Dcc ortho Ncc 20 Ncc+3 ortho	Good response to bifocals	Need to continue wearing bifocals	35	Dcc ortho Ncc 14 Ncc+3 ortho	40
11	F	5	Dcc 25 Ncc 25	R − 0.50 L − 0.25	60	BLRc 6.75	6	Dcc 20 Ncc 35	5.7	12	15	Dcc 2 ET Ncc 20 Ncc+3 2 ET	Good response to bifocals, later decompensated to basic ET	Underwent BMRc 5.00	43	Dcc ortho Ncc ortho	40
12	M	4	Dcc 40 Ncc 40	R − 6.86 L−5.63	140	BLRc 8.25	16	Dcc 10 Ncc 50	13.3	18	20	Dcc 20 ET Ncc 50 ET Ncc+3 20 ET	Partially corrected by bifocals	Underwent BMRc 5.00	45	Dcc ortho Ncc ortho	140
13	F	7	Dcc 20 Ncc 25	R − 0.75 L − 0.75	100	BLRc 6.25	1	Dcc 20 Ncc 35	4.0	27	2	Dcc 16 Ncc 30 Ncc+3 10	Partially corrected by bifocals	Underwent BMRc 5.25	31	Dcc ortho Ncc ortho	80
14	M	4	Dcc 25 Ncc 30	R + 0.25 L − 0.25	400	BLRc 7.25	4	Dcc 25 Ncc 35	5.7	39	4	Dcc 10 ET Ncc 25 ET Ncc+3 10 ET	Partially corrected by bifocals	Need to continue wearing bifocals	43	Dcc 8 ET Ncc 20 ET Ncc+3 6 ET	50

AC/A = accommodative convergence/accommodation; Adv = advancement; BLRc = bilateral lateral rectus recession; BMRc = bilateral medial rectus recession; D = diopters; Dcc = distance deviation with spectacle correction; DE = divergence excess; ET = esotropia; FU = follow up; LLR = left lateral rectus; LMR = left medial rectus; N-D = near-distance; NA = not available; Ncc = near deviation with spectacle correction; Ncc B = near deviation with lower segment of bifocal lens; No = number; ortho = orthotropia; Preop = preoperative; rec = recession; SA = stereoacuity; SE = spherical equivalent; SecArc = seconds of arc; XT = exotropia.

## Data Availability

The data presented in this study are available on request from the corresponding author. The data are not publicly available due to the institutional policy.
